# EEG Negativity in Fixations Used for Gaze-Based Control: Toward Converting Intentions into Actions with an Eye-Brain-Computer Interface

**DOI:** 10.3389/fnins.2016.00528

**Published:** 2016-11-18

**Authors:** Sergei L. Shishkin, Yuri O. Nuzhdin, Evgeny P. Svirin, Alexander G. Trofimov, Anastasia A. Fedorova, Bogdan L. Kozyrskiy, Boris M. Velichkovsky

**Affiliations:** ^1^Department of Neurocognitive Technologies, Kurchatov Complex of NBICS Technologies, National Research Centre “Kurchatov Institute,”Moscow, Russia; ^2^Department of Cybernetics, National Research Nuclear University MEPhIMoscow, Russia; ^3^Department of Computer Systems and Technologies, National Research Nuclear University MEPhIMoscow, Russia; ^4^Centre for Cognitive Programs and Technologies, Russian State University for HumanitiesMoscow, Russia; ^5^Department of Psychology, Technische Universität DresdenDresden, Germany

**Keywords:** brain-computer interfaces, human-computer interfaces, gaze interaction, assistive technology, eye tracking, detection of intention, slow cortical potentials, stimulus-preceding negativity

## Abstract

We usually look at an object when we are going to manipulate it. Thus, eye tracking can be used to communicate intended actions. An effective human-machine interface, however, should be able to differentiate intentional and spontaneous eye movements. We report an electroencephalogram (EEG) marker that differentiates gaze fixations used for control from spontaneous fixations involved in visual exploration. Eight healthy participants played a game with their eye movements only. Their gaze-synchronized EEG data (fixation-related potentials, FRPs) were collected during game's control-on and control-off conditions. A slow negative wave with a maximum in the parietooccipital region was present in each participant's averaged FRPs in the control-on conditions and was absent or had much lower amplitude in the control-off condition. This wave was similar but not identical to stimulus-preceding negativity, a slow negative wave that can be observed during feedback expectation. Classification of intentional vs. spontaneous fixations was based on amplitude features from 13 EEG channels using 300 ms length segments free from electrooculogram contamination (200–500 ms relative to the fixation onset). For the first fixations in the fixation triplets required to make moves in the game, classified against control-off data, a committee of greedy classifiers provided 0.90 ± 0.07 specificity and 0.38 ± 0.14 sensitivity. Similar (slightly lower) results were obtained for the shrinkage Linear Discriminate Analysis (LDA) classifier. The second and third fixations in the triplets were classified at lower rate. We expect that, with improved feature sets and classifiers, a hybrid dwell-based Eye-Brain-Computer Interface (EBCI) can be built using the FRP difference between the intended and spontaneous fixations. If this direction of BCI development will be successful, such a multimodal interface may improve the fluency of interaction and can possibly become the basis for a new input device for paralyzed and healthy users, the EBCI “Wish Mouse.”

## Introduction

A brain-computer interface (BCI) is a tool of control and communication without using muscles or peripheral nerves, through the use of brain signals only (Wolpaw et al., 2002). The lay public often expects that such tools should directly and effortlessly converts internal intents into actions in the outside physical world. In reality, however, BCIs provide much slower interaction comparing to traditional human-machine interfaces, such as mouse and keyboard computer input. Most of mental operations and states cannot be detected from the electroencephalogram (EEG) or other non-invasive brain signals on single-trial basis within seconds. A typical BCI does not recognize the user's whishes or action plans directly; to send a command, a user needs to execute one of a limited range of motor imagery, cognitive or perceptual tasks that can evoke recognizable brain signals. Such tasks are typically unrelated to the current activity and their use imposes additional mental load and decreases the fluency of BCI use. Invasive BCIs are promising, but high risks and costs associated with brain surgery require further efforts to make this technology acceptable even for severely paralyzed patients (Lahr et al., 2015; Bowsher et al., 2016; Waldert, 2016).

**Figure 1:**
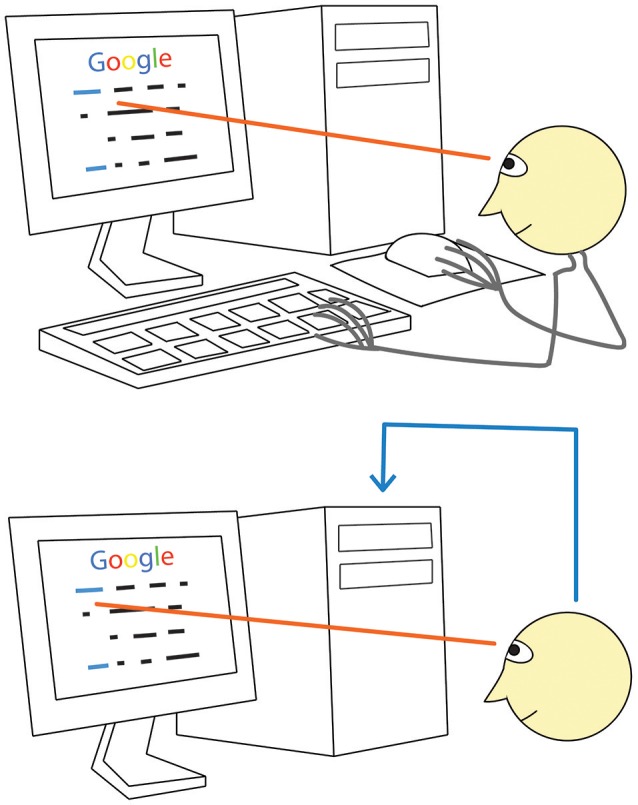
**A direct way to convey your desire to a computer: just look! When using a monitor, we normally look at a screen button or a link before clicking on them (upper panel)**. Between looking and clicking, we take the mouse (if we are not holding it all time), locate the cursor, move the cursor to the button or link, check if it reached the target. Gaze fixation at a given monitor location, however, can be promptly recognized with an eye tracker. If our intention to click would be recognized with some technology based on brain signal analysis (**lower panel, blue line**), computer control could be obtained without the above listed range of motor and sensory activities. (Generally, detection of gaze fixations alone is not enough due to the Midas touch problem, see text for details).

Can a machine recognize our intentions more directly at least in certain situations? Consider, for example, using a computer Graphical User Interface (GUI) by a healthy person. When he or she decides to click a screen button or a link, they need to take a mouse with their hand, move the cursor to the screen button or link and then click the mouse button (Figure 1, **upper panel**). Imagine that the computer analyzes his or her brain signals and can detect signal patterns related to the intention to make an action, so that the mouse is no longer needed. It is likely that, under these settings, the user could better focus on their main activity, because they would not need to switch to any motor task (Figure 1, **bottom panel**).

In this case, gaze positioning would play important role, and eye muscles need to be used intensively. However, the same is true for the operation of any non-invasive BCI when high information transfer rate is demonstrated: it is always a gaze-dependent operation, in a partial contradiction to Wolpaw et al. (2002) strict definition of a BCI. The user of such BCIs (the Steady State Visual Evoked Potential based BCI, SSVEP BCI; the codebook Visual Evoked Potential based BCI, cVEP BCI; the P300 wave based BCI, P300 BCI, when it is used by healthy people and some of the EEG electrodes are placed over the visual areas) selects one of a number of spatially distinct stimuli with his or her gaze, and the BCI task is actually to find where the user is looking by analyzing the brain's fully automatic response to stimulation.

In other words, these BCIs play the role of an eye tracker, and the brain's response to stimuli is used in such BCIs somewhat similarly to the use of the corneal reflection in a video based eye tracker. Moreover, a BCI that uses spatially distinct visual stimuli is an eye tracker with limited capabilities: unlike the usual eye trackers, it cannot determine coordinates of an arbitrary gaze position, but only indicate at which one of the stimuli the user is looking. Although attention is involved, and attempts were made to use overt and covert attention as separate BCI control channels, its direct (not gaze-mediated) effect is low comparing to gaze dependent effects. This is one of the main reasons why performance of the same BCI systems is, unfortunately, much lower in severely paralyzed users who do not control their gaze well (e.g., Sellers et al., 2010). In addition, as these BCIs depend on repetitive presentation of visual stimuli, GUIs should be significantly modified to become compatible with them.

The technology that enables direct interaction with computers using eye gaze also exists and is already used to assist paralyzed people in such tasks as typing and web browsing, with better performance than typical BCIs offer (Bolt, 1982; Jacob, 1990, 1991; Velichkovsky et al., 1997; Majaranta and Bulling, 2014). In a frequent variant of this technology, a video based eye tracker captures gaze direction. Intentionally prolonged gaze fixations (dwells) on elements of GUI, such as screen buttons, are detected and converted into equivalents of mouse clicks. It was, however, recognized early in the development of gaze-based interaction that the eye movements and fixations used for sending commands in such interfaces are similar to eye movements and fixations that are a part of normal eye behavior unrelated to interaction, such as viewing (Jacob, 1990, 1991). The interaction systems that are based on gaze direction, therefore, cannot distinguish between attempts to issue a command and the visual gaze fixations, and this leads to the Midas touch problem (Jacob, 1990, 1991): wherever the user looks, a command is activated, even if he or she has no intention to produce any command.

It was also stated at that early stage of developing gaze-base interaction with computers that the main issue in this field is “appropriate interaction techniques that incorporate eye movements into the user-computer dialogue in a convenient and natural way” (Jacob, 1990, p. 11). Such techniques have not been developed so far, despite many attempts to solve the Midas touch problem. For example, if only fixations on screen buttons exceeding a long (1000 ms or longer) threshold produce “clicks,” erroneous command activations will be rare, but the need to use such long fixation will slow down the control and make it tiresome. Impressive speed of interaction was recently achieved with dwell-free typing approach (Kristensson and Vertanen, 2012; Sarcar et al., 2013; Pedrosa et al., 2015), however, it seems to impose high attentional demands. Another recently developed approach to increase the speed is to use, as a marker of control, a saccade to a new position of interest that naturally occurs immediately after the feedback at the position where the user currently looks for control (Publicover et al., 2016). In this case, short fixation duration thresholds can be used because unintended selection are typically not confirmed by such well-timed saccades. This system, by definition, cannot work when the user does not respond to feedback quickly enough. Thus, attentional demands imposed by this system can also be high. Other attempts to provide markers for command-related gaze fixations or eye movements (see, e.g., Velichkovsky et al., 1997; Majaranta and Bulling, 2014, for reviews) possessed the same or other drawbacks.

One may expect that brain state and/or course of brain activity differs strongly during gaze fixations related to attempts to send a command and gaze fixations related to other activities, such as free viewing or mind wandering. A “click” on a fixated GUI element could be made on the basis of fast detection of brain activity patterns that correlate with the intention to activate a command (Figure 1, **bottom panel**). The joint use of the information from “eyes and brains” for GUI control (“point with your eye and click with your mind!”) was proposed as early as in 1996 (Velichkovsky and Hansen, 1996).

Several attempts to enhance gaze based control with a “mental click” recognized by BCI has been made. However, the use of additional mental tasks, such as motor imagery (e.g., in Zander et al., 2010) or mental concentration (Kim et al., 2015) does not seem to be effective, because BCIs cannot reliably recognize such activity within a fixation unless it is very long. Moreover, the use of additional mental task may itself interfere with the main mental activity. It is, therefore, not surprising that in a recent extensive review of hybrid BCIs only 3 of 55 reviewed journal papers addressed the Midas touch problem (Banville and Falk, 2016; see also other reviews on hybrid BCIs by Pfurtscheller et al., 2010; Müller-Putz et al., 2015). It seems that mechanistically combining gaze based technologies with existing BCIs cannot lead to the development of an efficient input system.

Zander and colleagues (Ihme and Zander, 2011; Protzak et al., 2013) proposed to make use of the EEG patterns that naturally accompany gaze based control. Their works were based on the “passive BCI” approach, i.e., the detection of brain signal patterns that naturally accompany the brain activities of interest, without the need from the user to do anything voluntarily to evoke these patterns (Zander and Kothe, 2011). The passive BCI approach seems to be exactly what is needed for detecting intentions in the most unobtrusive way, while gaze dwells effectively reveal the time intervals where the markers of intentions can be searched for in the brain signals. The EEG was collected during fixations intentionally used for control and during non-controlling fixations and successfully classified off-line (Ihme and Zander, 2011; Protzak et al., 2013). However, gaze dwell time thresholds were again quite long, 2000 ms in Ihme and Zander (2011) and 1000 ms in Protzak et al. (2013). Moreover, the participant task in both studies involved visual search, and the classification could rely mainly on fixation-related potential (FRP) components related to finding a target, rather than to intention to act.

When a target is searched for among a range of distractors, one may use the EEG potentials that accompany target detection, such as the P300 wave. Recent studies of the FRPs in the visual search tasks demonstrated that finding an object, area or position of interest can be detected using a combination of eye tracking and EEG (Healy and Smeaton, 2011; Kamienkowski et al., 2012; Brouwer et al., 2013; Kaunitz et al., 2014; Devillez et al., 2015; Finke et al., 2016; Ušćumlić and Blankertz, 2016; Wenzel et al., 2016). In particular, Finke et al. (2016) reported high performance, with area under ROC curve (AUC) of about 0.9, for classification of short target vs. non-target fixations. Similar phenomena could be a basis of classification in studies by Ihme and Zander (2011) and Protzak et al. (2013). This approach, however, is limited, because a click on a screen button or link is not always required immediately after they are found. In many applications, the user should be at least given an option to execute control in less automatic way. The P300 wave, which plays important role in this approach, can decrease and even vanish under many conditions, for example, when the target is always found at the same position. Classification of FRPs can be also related to the effects from planning a saccade to a new location of interest (Graupner et al., 2011; Nikolaev et al., 2013), but its possible use in BCI is limited for similar reasons. An effective general-purpose interface may include algorithms for supporting fast responses to just-found targets, but has to be primarily focused on more direct procedures of intention detection.

The current study followed the proposals of Zander and colleagues (Ihme and Zander, 2011; Protzak et al., 2013), i.e., it relied on finding EEG (FRP) markers for gaze dwells intentionally used for control. However, we aimed on investigating the possibility to find the FRP markers of controlling fixations when relatively short (500 ms) dwell time threshold is used. Fixations of this length are frequently observed in normal viewing, and do not require significant effort when they should be produced voluntarily. Special attention was paid to equalizing irrelevant factors that could confound the difference between the fixations used for control and spontaneous fixations. We took a straightforward approach of collecting the synchronized gaze and EEG data from the participants in a realistic scenario, when they played a game with their gaze only. The gaze controlled variant of a known, sufficiently engaging computer game was designed for this study, to make possible obtaining a large number of fixations of required duration, both intentionally used for control and spontaneous ones.

Results from a pilot study of the FRP markers of controlling fixations and summaries of the current work were presented at a few conferences (Shishkin et al., 2015, 2016b,c). This publication provides the detailed description of the approach, methodology and results.

## Methods

### Participants

The study was conducted in 8 healthy participants (age 21–48, mean 29, one female) in accordance with the Declaration of Helsinki and the local institutional regulations. All participants gave written informed consent. They had normal or corrected to normal vision. Two of them (participants 6 and 7) had no previous experience with gaze based control. The others already participated in a few pilot experiments with the same gaze controlled game as in this study.

### Apparatus and software used in online experiments

Electroencephalogram (EEG) was recorded at 500 Hz and 24 bit voltage resolution with an *actiCHamp* amplifier and *actiCAP* active electrodes (Brain Products, Germany) from 19 positions (Fz, F3, F4, Cz, C3, C4, Pz, P1, P2, P3, P4, POz, PO3, PO4, PO7, PO8, Oz, O1, and O2). More electrodes were placed in the posterior area than in the anterior one, because our pilot studies indicated that the marker for controlling fixations was likely to be found in the occipitoparietal region. Reference EEG electrodes were located at earlobes. Electrooculogram (EOG) was captured with the same amplifier and active electrode set. For horizontal EOG, electrodes were placed about 1 cm from the outer canthi. Vertical EOG was recorded with an electrode about 2 cm below the right eye and another one at Fp2 position (this location was preferred to more standard lower locations to avoid interference with the forehead rest). Ground electrode was located at AFz. Electrode impedances were kept below 20 KOhm. Gaze coordinates were recorded monocularly at 500 Hz sampling rate with the *EyeLink 1000 Plus* video based eye tracker (SR Research, Canada).

Recording of EEG and EOG, their synchronization with the eye gaze data, online processing of the eye gaze data and running the gaze controlled game were made with custom programs under *Resonance*, a framework for prototyping multimodal (hybrid) BCIs being developed by one of the authors of this study (Y.O.N.). For offline synchronization of the data, markers were sent from the eye tracker computer's parallel port to the EEG amplifier's TTL port in the beginning of each game. To control the stability of synchronization, this procedure was repeated in the end of the games (the time difference was typically no larger than two samples).

Dwells were detected online using a spatial (dispersion-based) criterion: gaze position range on each of X and Y coordinates should not exceed 2° for the specified dwell time (500 or 1000 ms, depending on condition). This criterion was chosen for two practical reasons: first, it could be easily implemented in the online software; secondly, it provided subjective experience of reliable control, according to our observations in the pilot experiments with the gaze controlled game used in this study. Several additional criteria had also to be met: no dwell detected in a 3 × 3° squared region centered around the previous click's position for previous 3000 ms, and no dwell detected in any position for 500 ms or 1000 ms (depending on condition). Each time all these criteria were met, a “click” was produced in the game (an event with the same effect as of a computer mouse click), and this time minus 500 or 1000 ms (for 500 or 1000 ms conditions, relatively) was designated as the beginning of the dwell event. The location of each “click” was computed as medians of X and Y coordinates of the gaze within the dwell time interval (excluding subintervals corresponding to the blinks reported by the eye tracker).

For simplicity, we call in this paper all the detected intentional and spontaneous dwell events “fixations.” Although the criteria for their detection were rather loose and, theoretically, small amplitude saccades and re-fixations could happen within the dwell interval, it seemed unlikely that they could bias our analysis results.

### The gaze controlled game

To collect the EEG during diverse, intensive and realistic gaze-mediated interaction with a computer, we designed a gaze controlled game *EyeLines*. It was based on a computer game *Lines* (or *Color Lines*) (“Color Lines,” in Wikipedia. Retrieved June 27, 2016, from http://en.wikipedia.org/wiki/ColorLines). Like the original game, the *EyeLines* is a simple computer puzzle game, with the goal to construct as many “lines” from colored balls as possible. The player is presented with a square board on which three colored balls appear in the beginning of the game. On each turn, the player has to move one ball to a free cell on the board. When the required or higher number of balls with the same color form a “full” line, either horizontal, vertical or diagonal, these balls disappear. If no such line is formed, three new balls randomly selected out of seven different colors are put on random cells. The game is over when the board is full.

**Figure 2:**
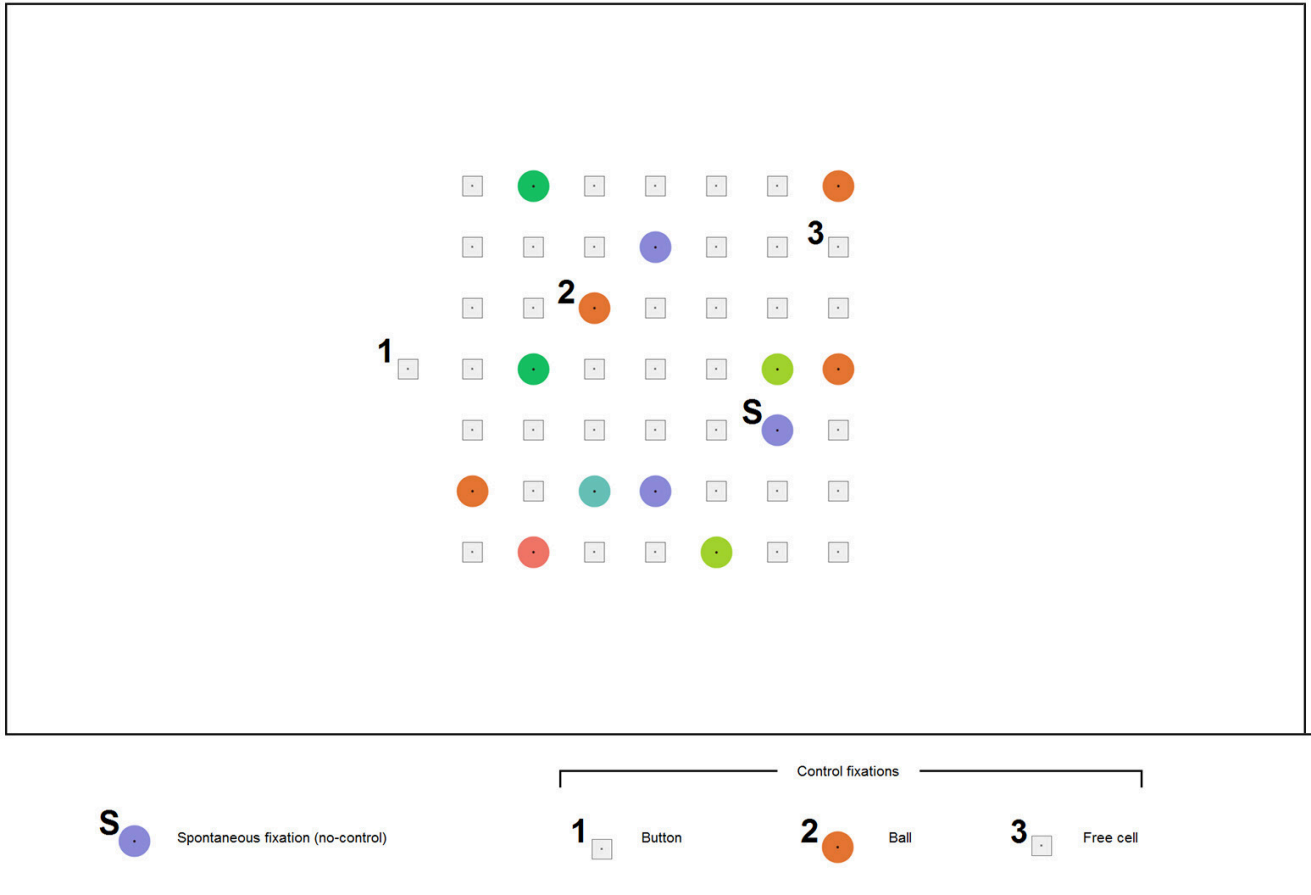
**An example screenshot of the ***EyeLines*** display**. The participants moved the color balls in order to construct lines from balls of the same color. Each move was made with gaze fixations only. A participant could make any number of spontaneous fixations (S) without any visible effect; however, their time was recorded. A dwell on the button (1) lead to appearing a ball in the button position, indicating that “control is on.” After this, a dwell on any ball in the game board (2) led to its “selection” (a frame appeared around it), and then the ball could be placed to a new position by a dwell on a free cell (3). (The digits 1, 2, 3, and the letter “S” were not a part of the actual display and were added to the screenshot for illustrative purposes).

In *Lines*, a move is typically made with a mouse click first on a ball and then on a free cell where it should be moved. In *EyeLines* (Figure 2), gaze dwells are used as an equivalent to mouse clicks, following the approach most often used in gaze controlled software. To avoid unwanted actions resulting from spontaneous long fixations and also to make possible collecting sufficient number of such fixations, gaze based control in the game board was off by default. After deciding which ball to move and to which free cell, a participant had to switch control on by gaze dwell on the only location where control was permanently on. This location was an additional cell (the *button*) positioned outside the game board either left, as in Figure 2, or right to it.

The participants were instructed to make the moves in the game by three subsequent *controlling fixations*: on the *button*, on a *ball* and on a *free cell* where the ball should be moved. A 500 or 1000 ms dwell time threshold was used (same in all games that constituted one experiment block) for all these actions. When fixation duration exceeded the threshold, a visual feedback was given. It was specific to each action: dwell on the button led to appearing, in this separate cell, a ball of color different from the board balls (the participants were told that this indicates switching the board control on); dwell on a ball within the board led to ball selection indicated by a gray square frame that appeared around the ball; dwell on a free cell led to moving the selected ball into this cell.

When control was off, fixations on the balls made no effect. They were considered as presumably spontaneous fixations (used for viewing or related to mind wandering) and constituted a separate type of *non-controlling fixations*.

To ensure more reliable gaze based control and to reduce complexity of the gameplay (so that even novices could make sufficient number of moves per game without long practice), we made some features of *EyeLines* simpler comparing to typical implementations of *Lines*. In particular, the board size was made 7 × 7 instead of 9 × 9 in ”classical” *Lines*, and the minimal number of balls required to form a full line was decreased from five to four. To support more detectable gaze fixations, a dot was put in the center of each ball and each free cell, the balls were drawn as plane circles (not as “3d like” spheres, to avoid asymmetrical features that could significantly shift the gaze from the center), and cells were designed as small squares with substantial spaces between them (Figure 2).

The game was presented on a 27″ LCD monitor positioned at the distance of 60 cm from participant's eyes. The game board subtended 18 × 18° on the monitor screen.

### Procedure

Participants were seated in a chair in front of the monitor. Chin and forehead rest was used to ensure stable head positioning. Participants were asked to relax and make no bodily movements during calibration and playing the game. They were instructed to make moves in the game by still looking sequentially at the button, the ball and the free cell, each time until they saw the feedback. After a move was completed, they could freely look at the board and fixate any ball or free cell for any time without evoking any action, until they dwelled at the button again for a time exceeding the threshold.

Participants were told to refrain from hurrying up with each move and to find “better” moves whenever possible, but not to think for too long time. In the preliminary experiments, however, we found that the *EyeLines* players often tend to makes moves as fast as possible even when they were urged not to do so. Due to this, sufficient number of non-controlling fixations could be not always collected. Therefore, an additional feature was introduced to *EyeLines*: after each 4–8 moves the cell outside the board that indicated the button's position disappeared from the screen for 10 s and the dwell on its former position had no effect, so that the participant could not make moves during this time. The participants were told that they had to use this time for planning the further moves. In addition to encouraging higher number of long fixations within this time, this trick presumably could help to prevent the participants from playing the game at too fast pace.

To those participants who had no prior experience with *EyeLines* or *Lines*, rules of the game were explained prior to EEG electrode mounting. They played two games with short duration (2 min) for practice.

In the main part of the experiment, all the participants played, in total, eight games with maximum duration of 5 min. The session was divided into two blocks of equal size, differed by the dwell time threshold, which was set at either 500 or 1000 ms. The order of the blocks was randomly assigned to the participants (three of the eight started with the 500 ms threshold block). The switch-on button position (left or right to the board) was alternated every game throughout the session. Its position in the first game was also chosen randomly (counterbalanced over the group). A 5 to 10 min rest was offered between the blocks, and shorter (typically up to minute) rest intervals were inserted between the games. Prior to each game, a nine-point calibration of the eye tracker was run.

### Offline data analysis

In this study, all EEG analysis, including classification, was made offline using the EEG data collected during the online gaze-based control. However, to make single-trial FRP classification performance evaluation realistic, the procedures for signal processing, feature extraction and application of the classifier to the new data were designed so that they could be used online without introducing any significant delay.

Signal preprocessing and segmentation were made mainly with *R*, while *MATLAB* (*MathWorks*) was used for visualization and classification. EEGLAB (Delorme and Makeig, 2004) routines *eegplot* and *topoplot* were used, relatively, for manual checking the quality of EEG/EOG epochs and for plotting scalp topographies. MANOVA was performed using *STATISTICA 7* (*StatSoft*).

Electroencephalogram (EEG) was re-referenced offline to linked earlobes, and bipolar EOG was calculated as the difference of amplitude in right/left and upper/lower channels, respectively. Line noise in EEG and EOG was reduced with 50 Hz notch filter (2nd order Butterworth). For creating the ERP time plots (but not for the other types of analysis), low-pass 7 Hz filter (2nd order Butterworth) was also applied.

For EEG and EOG analysis, [−500 +1500] ms epochs were extracted related to the start of four types of fixations exceeding the dwell time threshold: three types of controlling fixations (button, ball and free cell) and one type of non-controlling fixations. As non-controlling fixations, only fixations on balls made without fixating the button prior to them were considered; fixations of this kind on free cells were very rare and could not be used in the analysis. Data were analyzed separately for 500 and 1000 ms dwell threshold conditions. In some cases, they were also divided into subconditions related to left and right position of the button.

Fixation-related potentials (FRPs), by definition, are synchronized with the eye movements and, therefore, can be systematically contaminated by EOG artifacts. To deal with this problem, we set the baseline interval and borders of the EEG time intervals used for analysis and classification within the fixation. Our pilot studies suggested that the difference between the EEG amplitude in controlling and non-controlling fixations in the *EyeLines* game was most pronounced in the late part of fixation. In contrast, the amplitude of the lambda wave, the component of the fixation-related potentials with the latency of about 100 ms from fixation start, did not exhibit pronounced dependence on gaze-based control. To make the baseline less sensitive to the lambda wave and other visual components that could be irrelevant for control detection, we decided to use as the baseline 200–300 ms interval relative to the fixation start. No high-pass filter was used (the EEG amplifier used in this study allowed for DC recording), so the amplitude shifts related to eye movements could not influence the fixation interval through the transient response of the filter. Because our study was focused on the EEG features that could be used in fast online classification of the controlling and non-controlling fixations, the upper border of the analyzed interval was set at 500 ms both in 500 ms and 1000 ms dwell threshold conditions.

After baseline subtraction, we checked the number of epochs with amplitude higher than 70 μV in 200–500 ms interval in any channel, per fixation type, condition and participant. The highest incidence of such epochs was only five, while in most cases no such epochs were observed at all. Therefore, no artifact rejection procedure was applied to the data.

### Feature extraction and classification

Electroencephalogram (EEG) features were extracted from only 13 posterior channels (P1, P2, P3, P4, Pz, PO3, PO4, PO7, PO8, POz, O1, O2, and Oz). Due to low number of non-controlling fixations exceeding 1000 ms threshold, the classifier was trained using only the data from the condition where this threshold was used. Therefore, the right border of the interval for feature selection was set as the lowest dwell threshold in our experiment, i.e., 500 ms. (In online mode, not implemented in this study but supposed to be the main application of its results, a fixation exceeding this threshold should be classified as soon as possible, thus features could be taken only before the threshold.) The left border was set at 200 ms after the fixation start, to exclude the EOG artifacts. As in the EEG analysis described above, mean amplitude of the 200–300 ms baseline interval was subtracted from the EEG amplitudes channelwise, and no filter was used (except for the 50 Hz notch filter during EEG recording).

To capture the development of the negative wave, we used overlapping windows of 50 ms length and time step 20 ms (200–250, 220–270, and 440–490 ms, total *n* = 13 windows). The feature vector (169 features) for each fixation *k* was composed of the mean values of EEG amplitude *m*_*k*_*(i, c)* computed for each of these windows (*i* = 1–8) in each channel (*c* = 1–13).

One class of the data was the non-controlling fixations. The other class was formed either from the button fixations only (Trainset 1) or from all controlling fixations (Trainset 2). Trainset 1 was used because button FRPs differed most strongly from the non-controlling fixations, so their classification could provide an upper estimate of the performance. It is also possible that only the first fixation in a controlling sequence of fixations should be detected in the EBCI. Trainset 2 was used to explore how well the classifier could be used with different type of controlling fixations without additional tuning to each of them.

Two classification approaches were applied to the data separately. The first one was the Linear Discriminate Analysis (LDA) with shrinkage regularization (Blankertz et al., 2011) implemented in Fieldtrip toolbox for EEG/MEG-analysis (Oostenveld et al., 2011, http://www.ru.nl/neuroimaging/fieldtrip), further referred to as Shrinkage LDA. The second approach, the Committee of Greedy Classifiers, further referred to as Committee, was to create a pool of weak threshold classifiers performing in one-dimensional feature spaces (one classifier per feature), to select classifiers with a greedy algorithm and to use them in a classifier committee. More specifically, each weak classifier was trained independently on one of the 169 features, and a greedy algorithm was used to select 15 features and corresponding classifiers from the pool (at the first iteration, the classifier which had the minimal classification error was chosen to become a committee member; at each of further iterations, the classifier which minimized the error of committee was added to it). The Committee classified fixations by simple voting of the selected weak classifiers (Trofimov et al., 2015).

Thresholds for the classifiers were adjusted using validation subsets to obtain at least 0.90 specificity. The reason to aim on relatively high specificity in expense of sensitivity was that in EBCIs, i.e., interfaces based on combining gaze based control and the BCI technology, low sensitivity of an EEG classifier can be partly compensated by additional gaze based control routines. In particular, if users found that the interface does not respond to their intention as fast as they expect, they can simply dwell longer, so that the interface may recognize the intention without the EEG, just with an additional, longer dwell time threshold. In contrast, the specificity of the interface should be kept high in most cases, because too frequent false alarms can be annoying and even dangerous (e.g., in robotic applications).

For each classifier, performance was estimated separately for training and testing using either Trainset 1 or Trainset 2, and for testing on data from either 500 ms or 1000 ms dwell threshold conditions, to estimate if the same classifier could be used effectively for different types of data.

Five-fold cross-validation was used for estimation of the classifiers' performance, except for the cases when training and testing was done on different subtypes of fixations. Details on how the data were divided into training, validating and testing subsets are given in notes under relevant tables (**Tables 2–5**) in the *Results* section.

## Results

### Behavioral data

Each participant was able to play *EyeLines* with gaze control only, i.e., without any manual input. The control was very stable: participants reported that they encountered from 1 to 4 errors per session, including ball selections or free cell selection incorrectly recognized by the software, as well as their own errors. Offline analysis of gaze data showed that there were also rare cases when the participants dwelled on two or more balls in a row when control was on, or attempted to make a move that violated the rules and therefore was not actually executed; these cases were excluded from the analysis, except for some of the rarest cases which were difficult to separate due to technical reasons (the latter lead to slightly higher number of ball selections comparing to button activation events). On average, a participant made about 150 moves in the condition with 500 ms dwell threshold and about 120 moves in the condition with 1000 ms dwell threshold.

The number of controlling and non-controlling threshold-exceeding fixations collected in the experiment is given in Table 1. About 160 spontaneous fixations of 500 ms or longer duration were collected with 500 ms threshold, while the number of 1000 ms or longer spontaneous fixations was about 15 times lower. Except for these long spontaneous fixations, the number of collected fixations was sufficient for analysis, varying from 74 for free cell selection with 1000 ms threshold (in participant 2), to 208 for non-controlling fixation with 500 ms threshold (in the same participant). The number of controlling fixations was higher in every participant in the 500 ms compared to 1000 ms threshold conditions (see Table 1), possibly due to the restriction of the total game duration that was same in both conditions. However, the size of this difference was small in all participants (varying from 20 to 34%), therefore, no correction of the EEG analysis procedure was needed.

**Table 1:** **Number of controlling and non-controlling (spontaneous) fixations exceeding the dwell time threshold in the 500 ms and 1000 ms threshold conditions (collapsed over left-button and right-button conditions)**.

**Sbj**.	**500 ms fixation threshold**					**1000 ms fixation threshold**				
	**Non-controlling fixations**	**Controlling fixations**				**Non-controlling fixations**	**Controlling fixations**			
		**All**	**Button**	**Ball**	**Free cell**		**All**	**Button**	**Ball**	**Free cell**
1	114	444	150	148	146	9	369	124	123	122
2	208	364	120	128	116	15	271	101	96	74
3	144	408	138	139	131	13	325	109	108	108
4	200	549	184	184	181	24	446	150	148	148
5	162	435	147	148	140	4	338	113	113	112
6	124	527	176	180	171	5	428	145	142	141
7	123	442	151	153	138	7	358	122	121	115
8	194	517	171	175	171	9	378	125	127	126
Mean	158.6	460.8	154.6	156.9	149.3	10.8	364.1	123.6	122.3	118.3
Std.	38.0	64.1	21.2	20.4	22.7	6.5	56.0	16.9	17.1	22.6

### Overview of the fixation-related potentials (FRPs)

Our main interest was to characterize the EEG features that can be used for online detection of the dwell-based gaze control and, therefore, should appear earlier than the dwell time threshold. In the current study, for the sake of simplicity, we focused only on amplitude (not spectral and not synchrony based) features, on current fixation (not the preceding one) and only on the features that could not be affected by eye movement artifacts.

**Figure 3:**
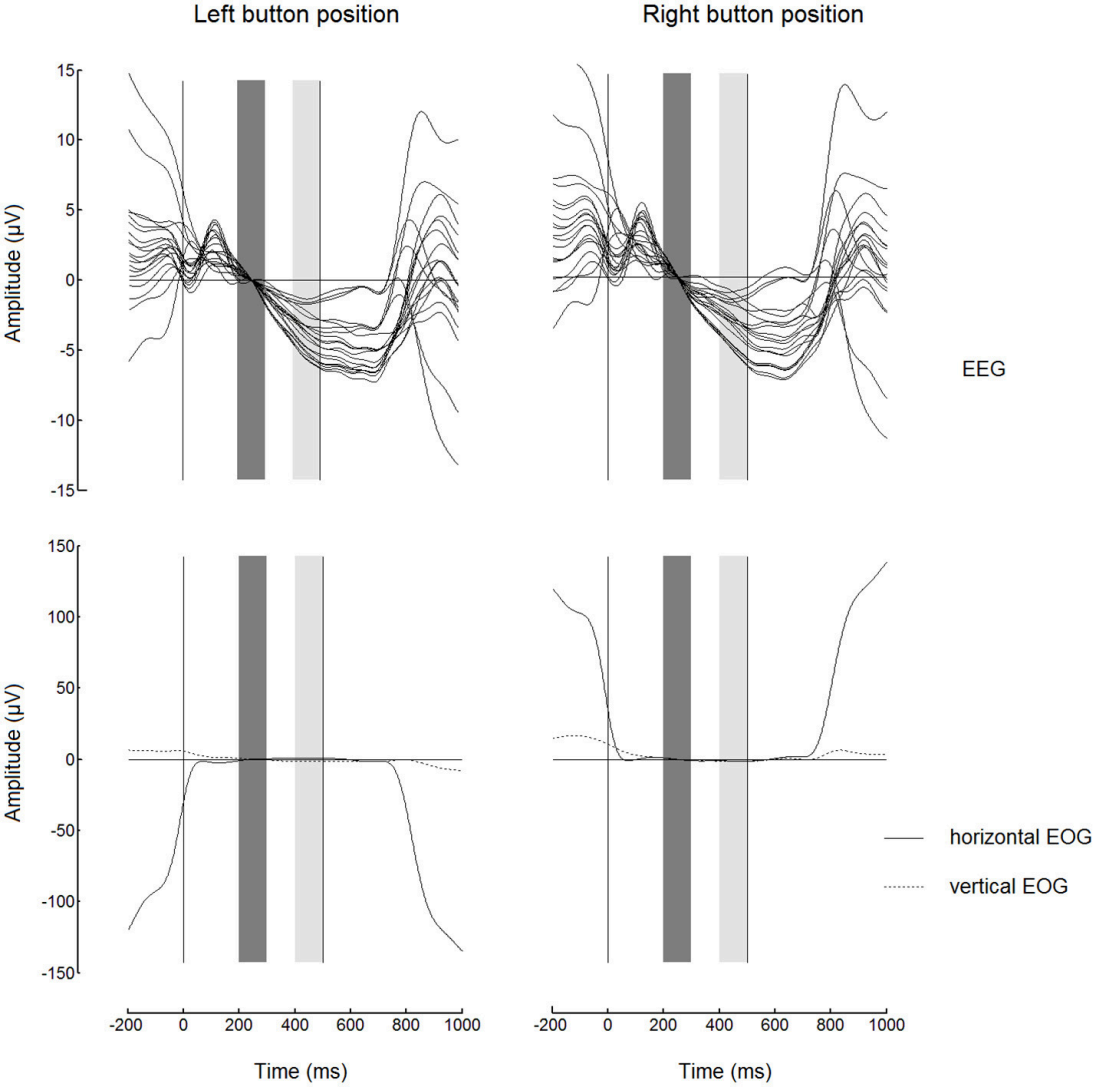
**Butterfly plots showing grand average (***n*** = 8) superimposed channels with fixation-related potentials (FRPs, upper panels) and corresponding electrooculogram (EOG, lower panels) for fixations on button**. The signals were low-pass filtered at 7 Hz and baseline corrected (high-pass filtering was not used). Note the similarity between the waveforms for left and right button positions (in the left and in the right, respectively) within most of the fixation interval. Zero millisecond corresponds to the beginning of fixation. The baseline interval (200–300 ms) is shown by dark gray bars. The light gray bars show 400–500 ms interval from which the EEG amplitudes were averaged to obtain estimates of the trend over the fixation used in the subsequent analysis (Figures 4, 5). Dwell time threshold was 500 ms (marked with the vertical lines in the right edges of the light gray bars).

Before starting to analyze the FRPs, we had to check if the EEG at the output of our preprocessing algorithm was sufficiently free from the EOG contamination. The highest contamination could be expected in the case of button fixations, when the gaze fixated an extreme position and the horizontal EOG could be added to the EEG signal especially strongly and systematically. Figure 3 shows FRPs (as a “butterfly plot”) and EOG for the button events averaged separately for left and right button positions. While the EOG contamination was evident in the FRP waveforms before the start of the fixation (0 on the time scale) and after approximately 250 ms following the dwell threshold at 500 ms (right vertical line), the horizontal EOG had very low amplitude in the 200–500 ms range. This interval was used for the analysis of EEG amplitude in both 500 and 1000 ms dwell time threshold conditions.

**Figure 4:**
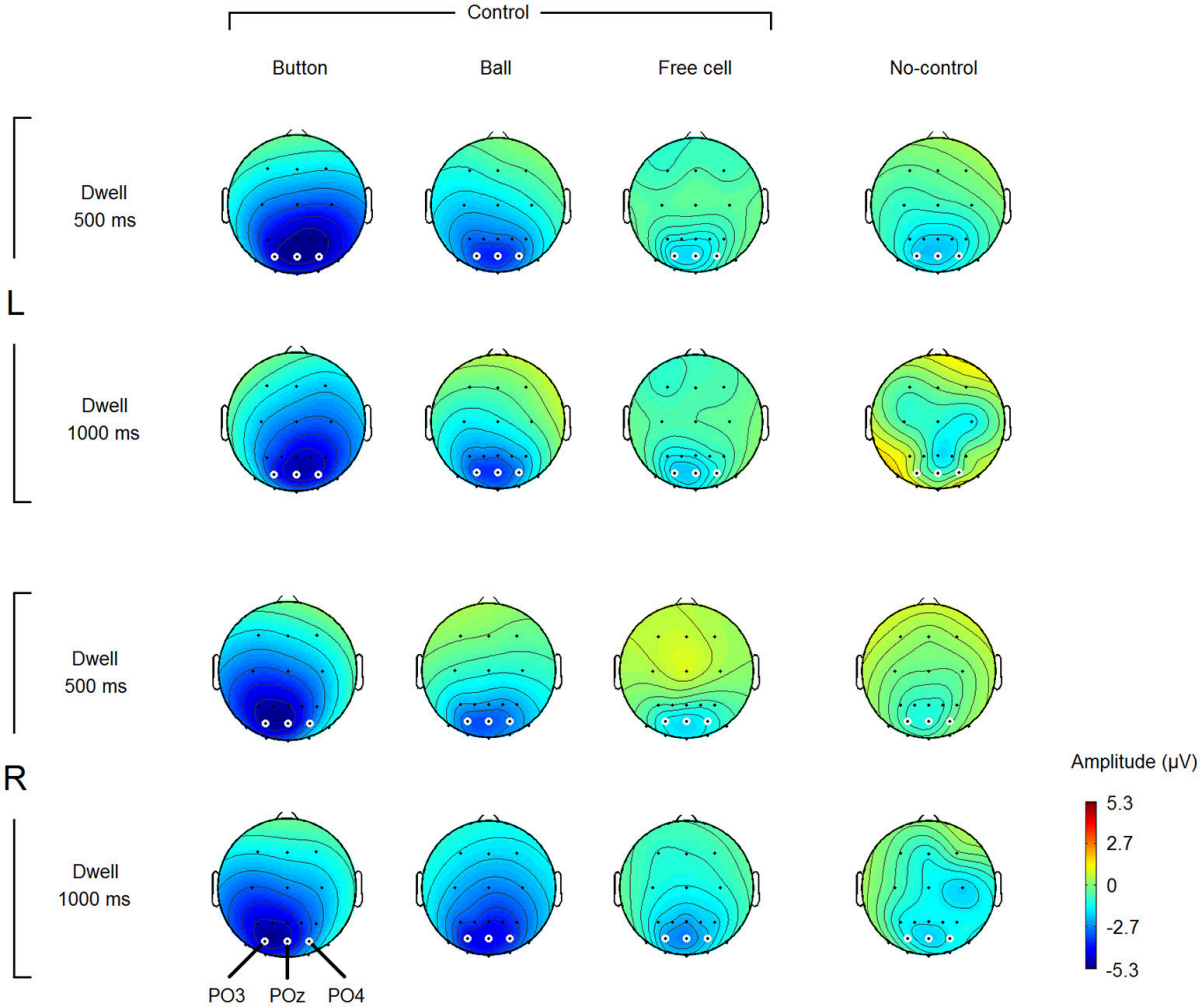
**Grand average (***n*** = 8) amplitude topography of the negative potential developed within different types of fixations and different dwell time thresholds**. L, left button position; R, right button position (note that the switch-on button was “clicked” not manually but by gaze fixations only). Amplitude estimates were obtained by averaging over 400–500 ms interval relative to fixation start (baseline 200–300 ms). Small white circles highlight the electrodes which were used in statistical analysis (POz, PO3, and PO4). Note that non-controlling fixations were rare in Dwell 1000 ms condition (see Table 1), thus the corresponding maps can be considered only as a rough estimate.

Figure 3 reveals a nearly linear negative trend in most of the EEG channels used in the study, possibly related to the expectation of the visual feedback (note that the experiment was organized in such a way that the gaze dwells intentionally used for control led to an equivalent of mouse click on the fixated position of the screen). Since we already used an interval near the start of this trend (200–300 ms) as the baseline, an average over an interval near the threshold can be used as a robust estimate of the trend. To view the topography of this negativity, we averaged EEG amplitude over 400–500 ms interval and plotted the results. Figure 4 shows grand average maps (individual maps had a similar pattern). According to this figure, the negativity was prominent in the parietooccipital area in all conditions. The maps also demonstrate a left-right asymmetry in Button condition that depended on button position: it was slightly stronger in the hemisphere contralateral to the gaze controlled switch-on button position where gaze dwelled in these fixations. Specifically, the difference between PO4 and PO3 amplitude in Button fixations with 500 ms dwell threshold was −0.4 ± 1.7 μV (M ± SD) in left button condition and +2.2 ± 1.3 μV in right button condition. The difference was significant, according to paired Student's *t*-test [t_(7)_ = −3.2, *p* = 0.02]. In Ball fixations, no difference between the two positions of the button was observed [the corresponding values were + 0.5 ± 0.9 μV and + 0.3 ± 0.8 μV, t_(7)_ = 0.5, *p* = 0.6].

Note that Button fixations were the most extreme fixations required for playing the game (the switch-on button was positioned left or right to the game board), while Ball fixations were all within the game board. The presence of the laterality effect in the Button but not in the Ball fixations means that it was not related to the direction of the previous saccade but is not sufficient to decide whether it was related to the current fixation or to the direction of the saccade to the next position that could be planned within this fixation. Nevertheless, the laterality effect is interesting because it was not reported so far for the Stimulus-Preceding Negativity (SPR) that could be the closest “relative” of the observed negative variation in the psychophysiological literature (see Discussion for more details).

In the rest of the amplitude analysis, POz electrode was used, because it the negativity that marked control-on fixations was typically highest at this location.

### Factors influencing the FRPs

Generally, FRPs may depend on many factors that may vary significantly during gaze interaction. These effects may negatively affect the stability of EBCI classifiers built using the FRPs or can be used for increasing the amplitude of the FRPs markers and, thus, for improving the EBCI performance. Detailed analysis of such effects could not be performed within the current study. However, we were interested to estimate if the negativity in the intentional fixation can be influenced by several factors which were well controlled in our experiment: left or right position of the switch-on “button” (which was already shown to produce a laterality effect), dwell time threshold (low effect of this factor would suggest that varying time of the feedback can be applied in online operation of EBCI likely without strong reduction of performance) and fixation type (it was most important to confirm the difference between spontaneous (non-controlling) and controlling fixations, but the difference between different types of controlling fixations should also be taken into account in EBCI development).

For this analysis, each participant's POz amplitudes averaged over 400–500 ms interval (with 200–300 ms baseline) for each fixation type and dwell time threshold were submitted to a 3-way MANOVA, with the following repeated measures factors:

- Button Position, with 2 levels: Left and Right,
- Dwell Threshold, with 2 levels: 500 ms and 1000 ms dwell time,
- Fixation Type, with 4 levels: Button, Ball, Free Cell, No-Control.

Significant effect was found for Fixation Type [Wilk's λ = 0.06, *F*_(3, 5)_ = 24.6, *p* = 0.002], whereas the Button Position effect [Wilk's λ = 0.96, *F*_(1, 7)_ = 0.32, *p* = 0.6], Dwell Threshold effect [Wilk's λ = 0.99, *F*_(1, 7)_ = 0.02, *p* = 0.9] and all interactions between factors were not significant. According to *post-hoc* Tukey HSD test, amplitude at POz in 400–500 ms interval was significantly more negative in Button comparing to No-Control fixations (*p* = 0.0002), in Ball comparing to No-Control fixations (*p* = 0.01) and in Button comparing to Free Cell fixations (*p* = 0.0007). In Ball fixations, POz was nearly significantly more negative than in Free Cell fixations (*p* = 0.064), while the difference between No-Control and Free Cell fixation as well as between Button and Ball fixations was not significant (*p* = 0.18 and *p* = 0.8, respectively).

**Figure 5:**
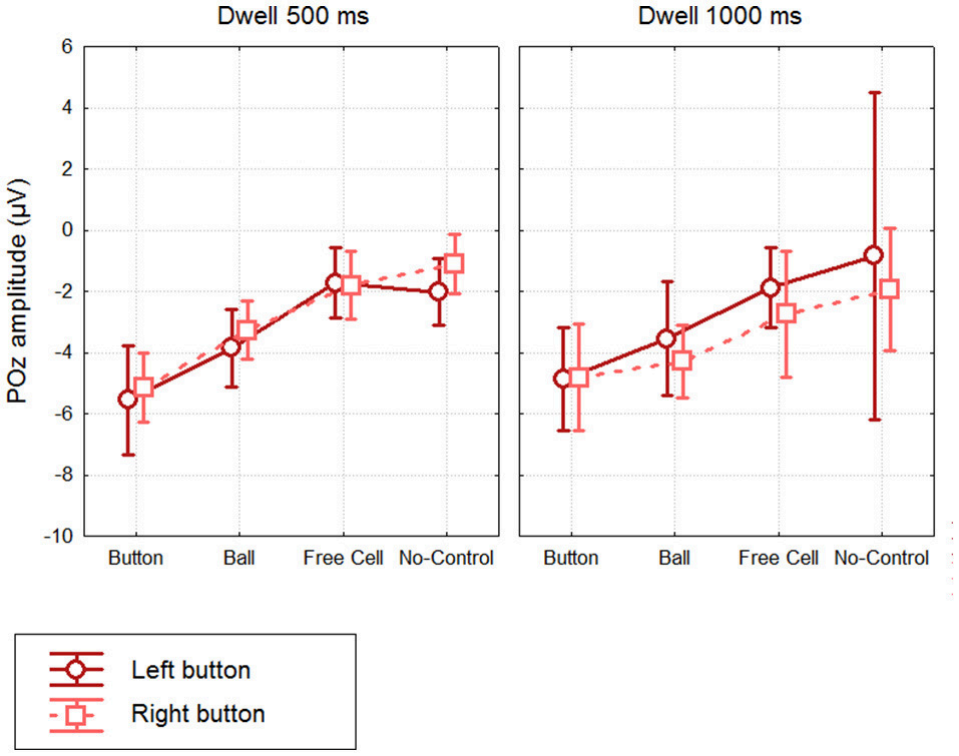
**Grand mean (***n*** = 8) values and 95% confidence intervals of the negative potential at POz developed within different types of fixations, using different dwell time thresholds and button positions**. Amplitude estimates were obtained by averaging over 400.500 ms interval relative to fixation start (baseline 200. 300 ms). Note that No-Control fixations were rare in Dwell 1000 ms condition (see Table 1), thus the average for these data can be considered only as a rough estimate.

Mean amplitudes and confidence intervals are shown in Figure 5. Individual data generally followed the same pattern, with largest POz amplitude in Button fixations. With 500 ms dwell threshold, POz amplitude ranged −3.6 to −10.3 μV in the Left button condition (−5.5 ± 2.1 μV) and −3.4 to −7.4 μV (5.1 ± 1.4 μV) in the Right button condition (the values in the parenthesis are M ± SD). POz amplitude in No-Control fixations, however, tended to be also negative (Left button: −0.4 to −4.0 μV, −2.0 ± 1.3 μV; Right button: +0.6 to −3.2 μV, −1.1 ± 1.2 μV).

**Figure 6:**
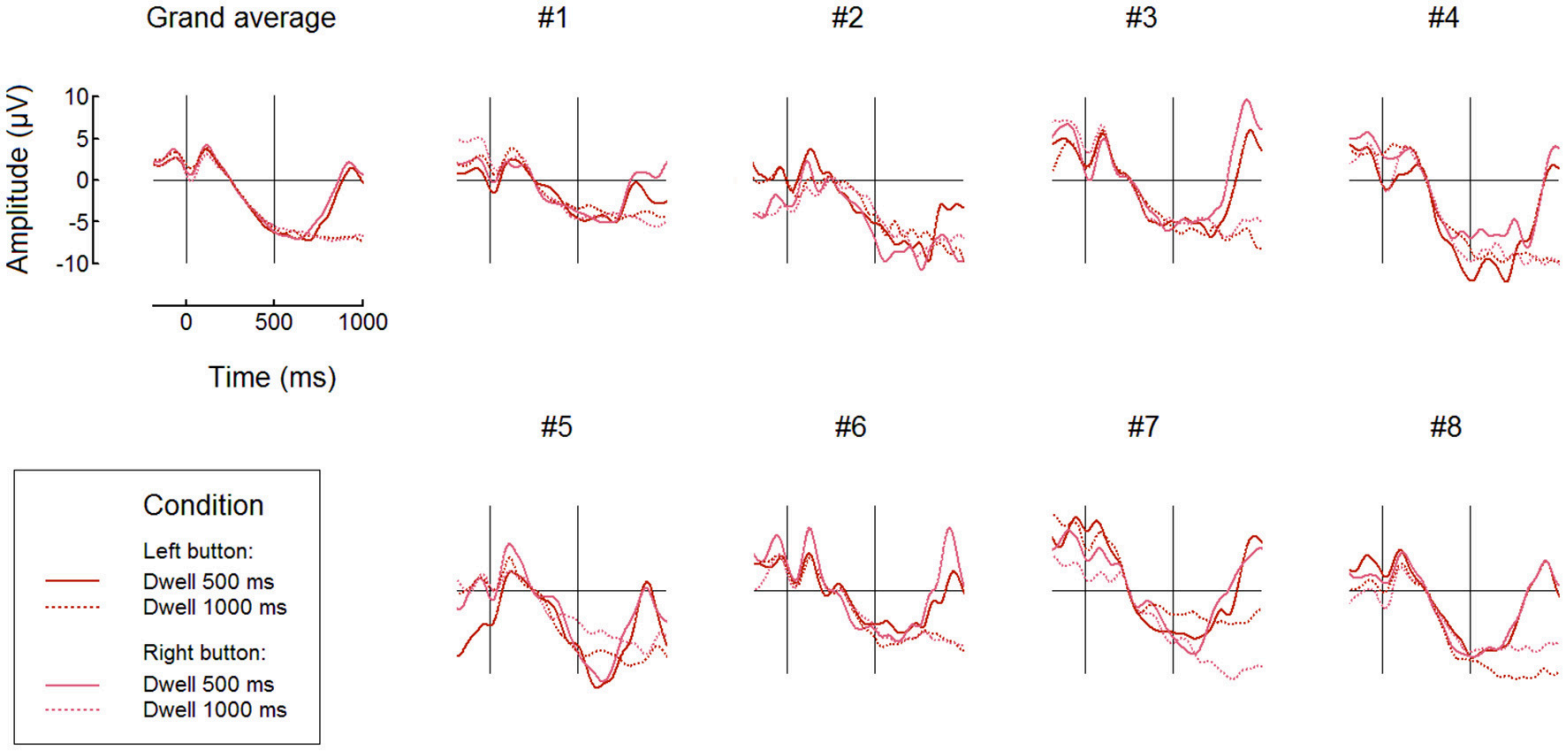
**Comparing different dwell time thresholds and button positions: grand average (***n*** = 8) and individual FRP waveforms observed at POz in Button fixations**. Fixation start and the 500 ms dwell time threshold are denoted by vertical lines. Filtering and baseline as in Figure 3.

Individual and averaged over the groups FRPs at POz for Button fixations and both 500 ms and 1000 ms thresholds are shown, separately for the left and right button conditions, in Figure 6. Note that in the 200–500 ms interval the signal pattern was similar for all the compared conditions, and the amplitudes and time courses were, in general, very similar in different dwell threshold and button position conditions (Figure 6). Importantly, during Button and Ball fixations the negativity at POz was developed over the first 500 ms in all participants (this was not the case only in the Free cell fixations for several participants).

**Figure 7:**
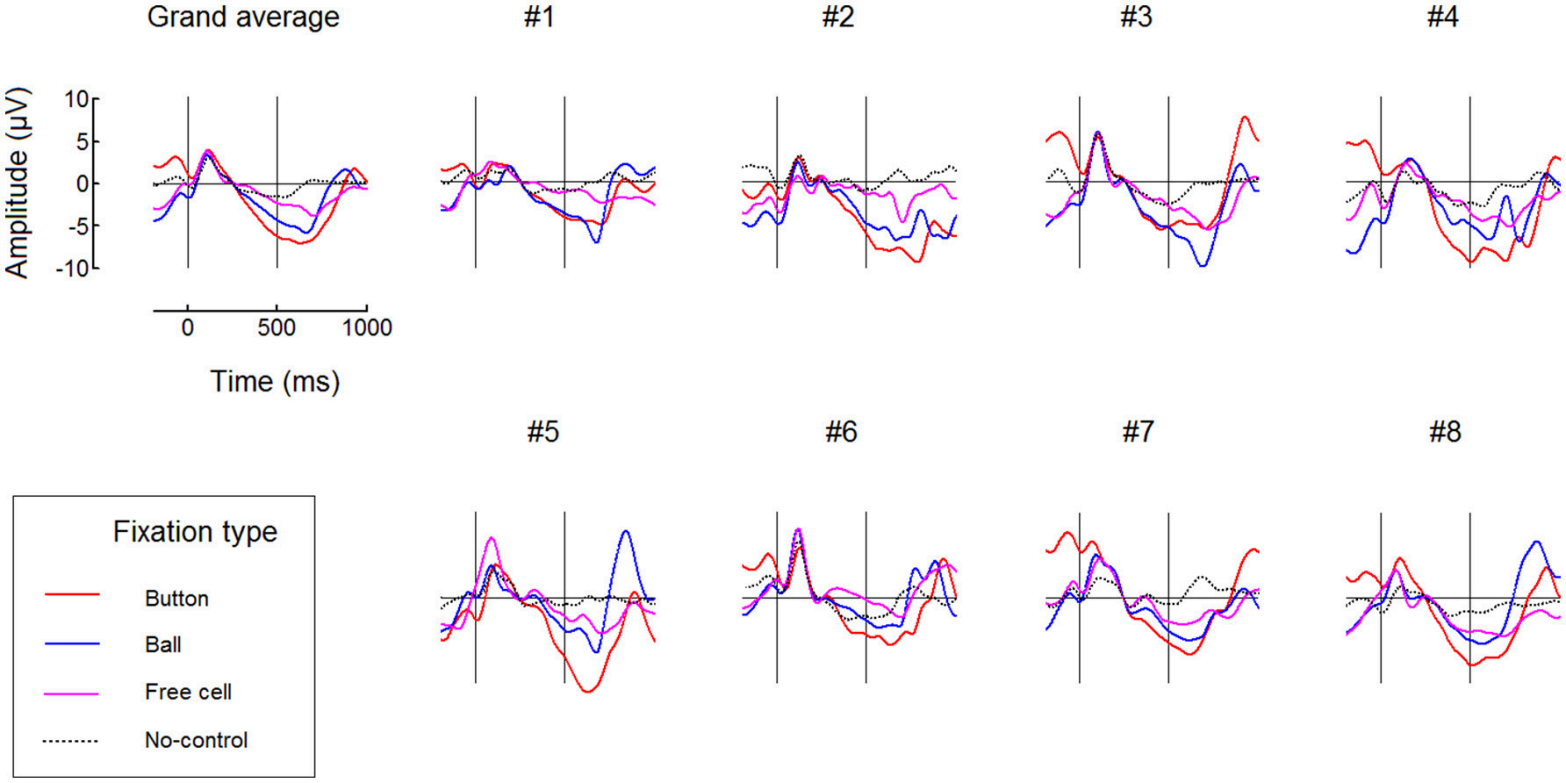
**Comparing different types of fixations: grand average (***n*** = 8) and individual FRP waveforms observed at POz with 500 ms dwell time threshold**. Here, left and right button data were averaged. Fixation start and the dwell time threshold are denoted by vertical lines. Filtering and baseline as in Figure 3.

FRPs time courses for all fixation types averaged over the left and right button positions are presented in Figure 7 (only for the 500 ms dwell threshold condition). This figure confirms the difference between fixation types: subsequent fixations were progressively weaker within the triplets used to make moves. More detailed analysis is needed to estimate possible impact of the negativity from previous fixation(s) on the baseline, that could, theoretically, cause this progressive decrease. Also, additional experiments and analysis are needed to rule out the possibility that it was an extreme eye position that enabled high effect in Button fixations and to rule out possible effects that the preceding high-amplitude saccade could have on it or (through its effect on the lambda wave) even on the beginning of the baseline (cf. Nikolaev et al., 2016).

Figure 7 shows that, although No-Control fixations were accompanied by the negativity at POz in some participants, its growth tended to terminate in these fixations earlier than in the controlling fixations. It was possible that the participants sometimes forgot to switch control on before fixating a ball and quickly recognized their fault but could made a saccade before the dwell time threshold, so such fixations were erroneously considered as non-controlling ones in our analysis.

### Classification

Finally, we estimated the possibilities to use the EEG marker of the gaze dwells used for intentional control by training statistical classifiers on the collected data and testing their performance offline under different settings that could be generally reproduced in online operation of the hybrid gaze interaction/BCI system (EBCI). Only 300 ms long EEG segments corresponding to fixations (200–500 ms relative to fixation onset) were used for single-trial classification.

The classifiers were trained using non-controlling fixations as the non-target class. The target class was represented either by Button fixations only (Trainset 1), or by all controlling fixations (Trainset 2). For the LDA classifier with the shrinkage regularization, performance results are provided in Table 2 (Trainset 1) and Table 3 (Trainset 2). For the committee of weak classifiers selected by the greedy algorithm, performance results are presented in Table 4 (Trainset 1) and Table 5 (Trainset 2).

**Table 2:** **Performance of the LDA classifier with shrinkage regularization trained using only fixations on button as target fixations (Trainset 1)**.

**Sbj**.	**Test on fixations with 500 ms threshold**					**Test on fixations with 1000 ms threshold**		
	**Specificity**	**Sensitivity**			**AUC**	**Sensitivity**		
		**Button**	**Ball**	**Free cell**	**Button**	**Button**	**Ball**	**Free cell**
1	0.87 ± 0.13	0.23 ± 0.17	0.05	0.12	0.64 ± 0.06	0.16	0.07	0.09
2	0.85 ± 0.10	0.29 ± 0.22	0.15	0.09	0.67 ± 0.09	0.28	0.08	0.05
3	0.84 ± 0.09	0.51 ± 0.24	0.26	0.29	0.76 ± 0.07	0.41	0.20	0.30
4	0.92 ± 0.12	0.29 ± 0.21	0.08	0.08	0.70 ± 0.08	0.30	0.05	0.11
5	0.83 ± 0.14	0.38 ± 0.11	0.20	0.29	0.77 ± 0.03	0.45	0.30	0.28
6	0.87 ± 0.09	0.23 ± 0.06	0.09	0.11	0.64 ± 0.07	0.19	0.12	0.13
7	0.93 ± 0.05	0.15 ± 0.09	0.03	0.02	0.57 ± 0.03	0.07	0.04	0.04
8	0.86 ± 0.11	0.43 ± 0.18	0.05	0.15	0.76 ± 0.04	0.34	0.13	0.10
Mean	0.87	0.31	0.11	0.14	0.69	0.28	0.12	0.14
Std.	0.11	0.20	0.08	0.10	0.09	0.13	0.09	0.10

*For training, recordings with 500 ms dwell threshold were used. On these recordings, 5-fold cross-validation was used to estimate, as M ± SD over folds, sensitivity and Area Under [ROC] Curve (AUC) for Button fixations, as well as specificity, in the 500 ms dwell threshold data. For each fold, the classifier was trained on 80% of the set, its threshold was adjusted on 8% aiming to obtain 90% specificity, and testing was done on 12%. In the case of testing on Ball and Free cell fixations, as well as for all 1000 ms dwell threshold data, the train and test sets were not overlapped and, therefore, the classifier was applied to all test data. Sensitivity is the same as True Positive Rate (TPR), here, the rate of correct classification of the intentional dwells. Specificity is equal to one minus the False Positive Rate (FPR); FPR is the same as the rate of false alarms, and in this work it corresponds to incorrectly classifying a spontaneous fixation as the controlling one (i.e., an intentional dwell)*.

**Table 3:** **Performance of the LDA classifier with shrinkage regularization trained using all controlling fixations as target fixations (Trainset 2)**.

**Sbj**.	**Test on fixations with 500 ms threshold**									**Test on fixations with 1000 ms threshold**		
	**Specificity**	**Sensitivity**				**AUC**				**Sensitivity**		
		**All**	**Button**	**Ball**	**Free cell**	**All**	**Button**	**Ball**	**Free cell**	**But-ton**	**Ball**	**Free cell**
1	0.90 ± 0.16	0.18 ± 0.19	0.21 ± 0.21	0.17 ± 0.18	0.18 ± 0.18	0.63 ± 0.06	0.64 ± 0.10	0.61 ± 0.05	0.64 ± 0.06	0.13	0.09	0.08
2	0.85 ± 0.07	0.28 ± 0.10	0.34 ± 0.17	0.30 ± 0.08	0.20 ± 0.13	0.63 ± 0.02	0.66 ± 0.06	0.64 ± 0.02	0.58 ± 0.05	0.30	0.24	0.16
3	0.96 ± 0.05	0.19 ± 0.12	0.24 ± 0.21	0.15 ± 0.10	0.15 ± 0.11	0.63 ± 0.04	0.70 ± 0.10	0.60 ± 0.06	0.59 ± 0.08	0.20	0.10	0.09
4	0.83 ± 0.17	0.32 ± 0.18	0.41 ± 0.19	0.26 ± 0.21	0.27 ± 0.17	0.64 ± 0.02	0.69 ± 0.07	0.62 ± 0.03	0.60 ± 0.03	0.34	0.24	0.32
5	0.83 ± 0.09	0.21 ± 0.08	0.25 ± 0.14	0.19 ± 0.08	0.21 ± 0.11	0.64 ± 0.03	0.67 ± 0.03	0.61 ± 0.03	0.66 ± 0.05	0.19	0.16	0.14
6	0.70 ± 0.25	0.32 ± 0.23	0.36 ± 0.27	0.30 ± 0.23	0.37 ± 0.28	0.59 ± 0.06	0.59 ± 0.06	0.56 ± 0.05	0.63 ± 0.07	0.31	0.21	0.27
7	0.90 ± 0.09	0.12 ± 0.09	0.19 ± 0.11	0.12 ± 0.06	0.13 ± 0.11	0.56 ± 0.01	0.60 ± 0.02	0.55 ± 0.03	0.56 ± 0.05	0.07	0.07	0.09
8	0.92 ± 0.05	0.26 ± 0.16	0.24 ± 0.15	0.28 ± 0.15	0.30 ± 0.19	0.71 ± 0.06	0.68 ± 0.07	0.72 ± 0.05	0.74 ± 0.06	0.26	0.27	0.21
Mean	0.86	0.24	0.28	0.22	0.23	0.63	0.65	0.61	0.63	0.23	0.17	0.17
Std.	0.15	0.17	0.08	0.07	0.19	0.04	0.04	0.05	0.06	0.09	0.08	0.09

*For training, recordings with 500 ms dwell threshold were used. On these recordings, 5-fold cross-validation was used to estimate, as M ± SD over folds, all the performance indices. For each fold, the classifier was trained on 80% of the set, its threshold was adjusted on 8% aiming to obtain 90% specificity, and testing was done on 12%. In the case of testing on 1000 ms dwell threshold data, the train and test sets were not overlapped and, therefore, the classifier was applied to all test data*.

**Table 4:** **Performance of the committee of 15 greedy classifiers trained using only fixations on button as target fixations (Trainset 1)**.

**Sbj**.	**Test on fixations with 500 ms threshold**				**Test on fixations with 1000 ms threshold**		
	**Specificity**	**Sensitivity**			**Sensitivity**		
		**Button**	**Ball**	**Free cell**	**Button**	**Ball**	**Free cell**
1	0.92 ± 0.04	0.31 ± 0.08	0.16	0.14	0.23	0.24	0.13
2	0.92 ± 0.03	0.40 ± 0.16	0.17	0.12	0.17	0.17	0.16
3	1.00 ± 0.00	0.20 ± 0.06	0.06	0.02	0.07	0.05	0.06
4	0.83 ± 0.04	0.44 ± 0.08	0.28	0.18	0.36	0.27	0.17
5	0.85 ± 0.06	0.45 ± 0.10	0.20	0.17	0.31	0.19	0.17
6	0.90 ± 0.06	0.39 ± 0.12	0.14	0.14	0.20	0.13	0.11
7	0.86 ± 0.06	0.47 ± 0.16	0.33	0.25	0.34	0.31	0.27
8	0.95 ± 0.04	0.41 ± 0.06	0.17	0.17	0.35	0.13	0.07
Mean	0.90	0.38	0.19	0.15	0.25	0.19	0.14
Std.	0.07	0.14	0.08	0.07	0.10	0.08	0.07

*For training, recordings with 500 ms dwell threshold were used. On these recordings, 5-fold cross-validation was used to estimate, as M ± SD over folds, sensitivity for Button fixations and specificity in the 500 ms dwell threshold data. For each fold, the classifier was trained on 80% and tested on 20% of the set. In the case of testing on Ball and Free cell fixations, as well as for all 1000 ms dwell threshold data, the train and test sets were not overlapped and, therefore, the classifier was applied to all test data*.

**Table 5:** **Performance of the committee of 15 greedy classifiers trained using all controlling fixations as target fixations (Trainset 2)**.

**Sbj**.	**Test on fixations with 500 ms threshold**					**Test on fixations with 1000 ms threshold**		
	**Specificity**	**Sensitivity**				**Sensitivity**		
		**All**	**Button**	**Ball**	**Free cell**	**Button**	**Ball**	**Free cell**
1	0.92 ± 0.08	0.22 ± 0.02	0.28 ± 0.07	0.22 ± 0.05	0.15 ± 0.03	0.25	0.27	0.17
2	0.91 ± 0.05	0.24 ± 0.08	0.30 ± 0.06	0.23 ± 0.09	0.18 ± 0.12	0.21	0.17	0.14
3	0.96 ± 0.04	0.18 ± 0.03	0.18 ± 0.04	0.19 ± 0.05	0.18 ± 0.09	0.11	0.10	0.12
4	0.91 ± 0.04	0.20 ± 0.02	0.30 ± 0.06	0.19 ± 0.01	0.10 ± 0.05	0.21	0.20	0.09
5	0.93 ± 0.06	0.26 ± 0.06	0.23 ± 0.11	0.24 ± 0.06	0.30 ± 0.09	0.22	0.24	0.19
6	0.93 ± 0.03	0.33 ± 0.06	0.34 ± 0.12	0.29 ± 0.04	0.36 ± 0.09	0.24	0.19	0.32
7	0.92 ± 0.08	0.26 ± 0.04	0.31 ± 0.11	0.27 ± 0.02	0.19 ± 0.06	0.29	0.21	0.17
8	0.84 ± 0.06	0.45 ± 0.07	0.50 ± 0.10	0.42 ± 0.12	0.43 ± 0.09	0.46	0.39	0.37
Mean	0.91	0.27	0.31	0.26	0.24	0.25	0.22	0.20
Std.	0.07	0.10	0.09	0.07	0.13	0.10	0.08	0.10

*For training, recordings with 500 ms dwell threshold were used. On these recordings, 5-fold cross-validation was used to estimate, as M ± SD over folds, all the performance indices. For each fold, the classifier was trained on 80% and tested on 20% of the set. In the case of testing on 1000 ms dwell threshold data, the train and test sets were not overlapped and, therefore, the classifier was applied to all test data*.

As follows from Tables 2–5, the procedure for setting the classifier threshold using the validation subset provided a good approximation to the target specificity value of 0.90. Insufficient number of the non-controlling fixations in the 1000 ms dwell threshold condition (see Table 1) made calculation of specificity for this condition impossible. For the 500 ms dwell threshold data and LDA classifier, specificity was almost the same for Trainset 1 and Trainset 2: M ± SD was 0.87 ± 0.11 and 0.86 ± 0.15, respectively. A similar pattern was observed in the case of the committee of classifiers, but the mean values were slightly higher comparing to LDA: 0.90 ± 0.07 and 0.91 ± 0.07, respectively, for Trainset 1 and Trainset 2. Since the average number of non-controlling fixations in 4 games with the 500 ms dwell threshold was about 160 (Table 1) and one game lasted 5 min or (rarely) slightly less, the rate of 500 ms or longer non-controlling fixations was about 160/(4 × 5) = 8 min^−1^, and the specificity of 0.9 corresponded to 8×(1–0.9) = 0.8 false alarms per minute.

**Figure 8:**
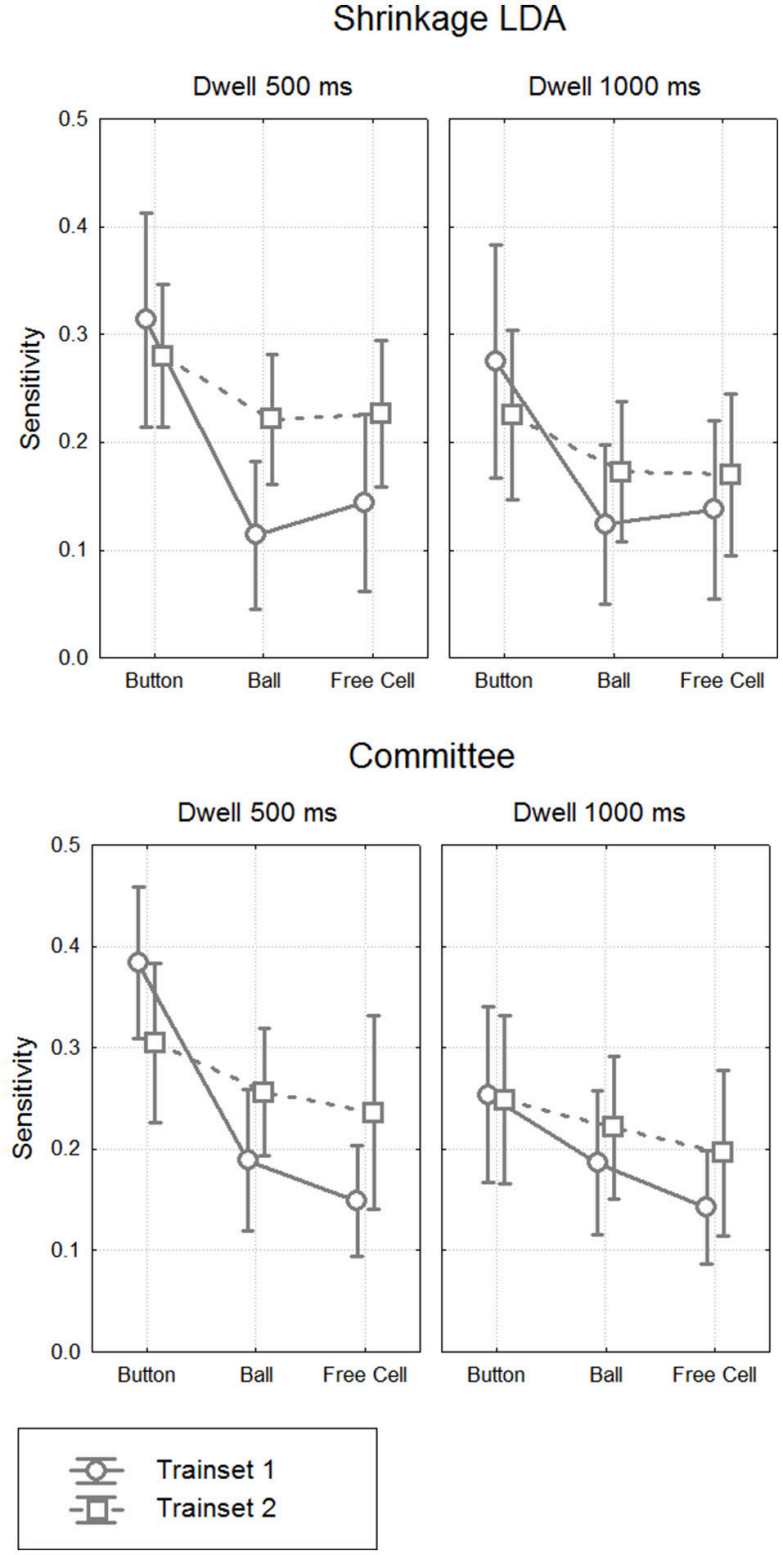
**Classifier sensitivity on the test data. Grand mean (***n*** = 8) values and 95% confidence intervals for different classifiers, different types of fixations and different dwell time thresholds**. The target (controlling) class in the train data was either button fixations (Trainset 1) only or all controlling fixations (Trainset 2).

It was not possible to compare the two classifiers using ROC AUC values (the index which integrates performance estimates for different thresholds), because they could be computed not in all cases. However, given that the specificity values did not strongly differ between the classifiers and the training sets, we decided to compare the sensitivity values directly between different settings (Figure 8). We run a single 4-way MANOVA analysis on all sensitivity data, with the following repeated measures factors:

- Classifier, with 2 levels: Shrinkage LDA, Committee of Greedy Classifiers,
- Training Set, with 2 levels: Trainset 1 (only fixations on button as controlling fixations), Trainset 2 (all controlling fixations),
- Threshold, with 2 levels: 500 ms dwell time, 1000 ms dwell time,
- Fixation Type, with 3 levels: Button, Ball, Free Cell.

MANOVA results are presented in Table 6. They generally followed the effects observed in the analysis of EEG amplitudes reported above, such as significant main effect for Fixation Type factor (sensitivity was significantly higher for Button fixations, according to *post-hoc* analysis). Main effect and almost all interactions were not significant for Classifier, except for Classifier × Training Set × Dwell Threshold interaction (*p* = 0.04). There was a tendency of better sensitivity provided by the committee of classifiers comparing to the LDA in the case of training on all types of fixations and applying the classifier to 1000 ms data (*p* = 0.08, *post-hoc* Tukey test).

**Table 6:** **Four-way MANOVA results for sensitivity**.

*Factor*	*Wilks' λ*	*F*	*df*	*p*
Classifier	0.90	0.80	1, 7	0.40
Training Set	0.86	1.15	1, 7	0.32
**Dwell Threshold**	**0.12**	**52.06**	**1, 7**	**0.0002**
**Fixation Type**	**0.06**	**44.68**	**2, 6**	**0.0002**
Classifier × Training Set	1.00	0.01	1, 7	0.94
Classifier × Dwell Threshold	0.87	1.05	1, 7	0.34
**Training Set** × **Dwell Threshold**	**0.46**	**8.23**	**1, 7**	**0.024**
Classifier × Fixation Type	0.54	2.55	2, 6	0.16
**Training Set** × **Fixation Type**	**0.23**	**10.18**	**2, 6**	**0.012**
**Dwell Threshold** × **Fixation Type**	**0.06**	**44.71**	**2, 6**	**0.0002**
**Classifier** × **Training Set** × **Dwell Threshold**	**0.52**	**6.34**	**1, 7**	**0.040**
Classifier × Training Set × Fixation Type	0.47	3.45	2, 6	0.10
Classifier × Dwell Threshold × Fixation Type	0.53	2.68	2, 6	0.15
**Training Set** × **Dwell Threshold** × **Fixation Type**	**0.35**	**5.62**	**2, 6**	**0.042**
Classifier × Training Set × Dwell Threshold × Fixation Type	0.77	0.91	2, 6	0.45

*Significant effects (p < 0.05) are shown in bold. Factors and their levels are described in the text*.

Although main effect was not significant for Training Set (*p* = 0.32), its interaction with Fixation Type was significant (*p* = 0.012). *Post-hoc* analysis revealed that the classifiers that were trained using all controlling fixations (Trainset 2) overperformed the classifiers trained on Button fixations only (Trainset 1) in the case of Ball and Free Cell fixations (*p* = 0.005 for both of them). With Trainset 1, sensitivity for the fixations not used for training (Button and Free Cell) was close to random level, especially with LDA classifier (Table 2), while improvement for Button fixations in the case of Trainset 1 did not reached statistical significance (*p* = 0.09, *post-hoc* Tukey test).

As one could expect, the classifier was more sensitive when the train and test data were recorded with the same dwell time threshold (*p* = 0.0002 for main effect of the factor Threshold). However, as follows from Tables 2, 3, the average difference was relatively small (on average, not higher than 0.03 for Trainset 1 and not higher than 0.06 for Trainset 2).

## Discussion

This study, for the first time, demonstrated that gaze fixations used for the interaction with a computer can be differentiated from spontaneous fixations using EEG markers from short time intervals (here, 200–500 ms relative to fixation start). This result opens the perspective of developing a hybrid “eye-brain computer interface,” the EBCI, as an unobtrusive communication and control tool both for paralyzed patients with preserved gaze control and for healthy users.

In addition, with special measures taken to obtain the fixation-related EEG potentials (FRPs) free from contamination with EOG artifacts, we revealed and quantitatively characterized a pattern differentiating the controlling and no-controlling (spontaneous) fixations in each of the eight participants.

The analysis of FRPs is challenging due to the systematic contamination of the EEG from electrical potential shifts accompanying the eyeball movements. Such contamination cause problems either when the EOG artifacts appear in the time intervals that are the main focus of analysis or when they are present during intervals used for baseline correction (Finke et al., 2016; Nikolaev et al., 2016). In addition, EEG components within the first 200 ms after fixation onset depend on the size of saccade preceding the fixation (Nikolaev et al., 2016). In our study, only the 200–500 ms interval relative to fixation start was used for FRP analysis and classification of fixations to avoid any interference. Moreover, we did not filter out low frequencies, to avoid contamination that can extend into this interval due to strong transient response after saccade-related shift of the EOG potential, and set the baseline intervals borders within the analyzed interval, i.e., at 200–300 ms relative to fixation onset. Positioning of the baseline in this interval means that results should be interpreted with care taking into account possible dampening of the activity of interest due to its presence in the subtracted signal. However, for demonstrating the possibility of developing an EBCI it was more important to prove that we obtained an EEG marker that was free from EOG contamination. As Figure 3 shows, the EOG contamination indeed was absent in the 200–500 ms interval of the analyzed data that could be affected by it most strongly, i.e., in Button fixations.

### The negative EEG wave

The topography of the EEG amplitudes in the 400–500 ms interval relative to fixation onset revealed a negativity focused at POz (Figure 4). Surprisingly, it was already developed in this interval even when the dwell threshold was 1000 ms. In the first and second of the three dwells used to make a move in the game (Button and Ball fixations), the EEG negativity at POz was strong in the 400–500 ms interval in all participants and significantly differed from the values in long non-controlling (spontaneous) fixations.

The EEG negativity that was characteristic for the fixations used for control in our study was growing along the course of fixations, while Ihme and Zander (2011) and Protzak et al. (2013) reported EEG markers (also in the form of negativity) early within the controlling fixations. This difference could be caused by the differences in study design and processing approaches. In particular, the FRP in the study by Ihme and Zander (2011) could be influenced by EOG (although it unlikely influenced the classification results, according to the classifier weight scalp maps), while Protzak et al. (2013) focused on a time interval that immediately followed finding the target by the participants and could contain related activity. However, the FRPs presented in the latter work also showed, in the later part of the fixation intervals (mostly not used for classification), a deflection in negative direction, that could resemble the negative wave observed in our study if the authors would choose the baseline in the same time interval as we did it.

When a fixation is intentionally used for sending a command to computer, a user is expecting a feedback signal. In such situations, Stimulus Preceding Negativity (SPN) is observed. This EEG phenomenon can have a form of sustained negativity in anterior areas (unlikely to be detected with our baseline) and a sharp growth of negativity in parietal areas (Brunia, 1988; Brunia and Van Boxtel, 2001; Van Boxtel and Böcker, 2004; Brunia et al., 2011; Kotani et al., 2015). However, in our study the negativity was shifted to the hemisphere contralateral to the fixated location in the condition where the gaze was strongly shifted to the left or to the right (dwell on the switch-on button), an effect not known for SPN. Saccade preparation (possibly accompanying Button and Ball fixations, and, in some cases, even Free cell fixations) is characterized by a similar negative wave in the parietal area and is stronger in the hemisphere contralateral to saccade direction (Klostermann et al., 1994; Berchicci et al., 2012; Krebs et al., 2012). However, in our experiment the saccades following dwell on the button should be made in the direction opposite to the fixation location, i.e., the negativity had higher amplitude in the hemisphere ipsilateral to the direction of the saccade that followed the dwell; one may hypothesize that the mechanism enabling an intentional fixation at some location may have something in common with planning a saccade to this location, but we failed to find an evidence for this in the literature. Other slow negative EEG components related to preparation for action, Contingent Negative Variation (CNV) and Readiness Potential (RP), have more anterior localization.

Thus, we could not reliably identify the nature of the negative wave that marked the intentionally used gaze fixations, and additional studies are needed to clarify this question. In particular, it will be of practical importance to understand if the amplitude decreases within the controlling triplets (Button–Ball–Free cell) was just the order effect (the feedback for the second and especially third fixations in the sequence could be less informative, or affected by a refractory effect, etc.), or it resulted from factors specific to gaze position and object properties. Note, however, that when the classifier was trained on all types of data its performance was nonrandom even for Free cell fixations. If the EEG markers of intention to act will turn to be sensitive to various factors (properties of fixations, fixated objects, visual context, previous fixations, previous or planned saccades, variations in difficulty of maintaining a fixation intentionally, etc.; see, e.g., Nikolaev et al., 2016), different classifiers could be used for each specific case.

### EBCI performance

To enable fluent interaction, an EBCI should use short EEG segments. Our results demonstrated, for the first time, that EEG segments as short as 300 ms were sufficient to detect gaze fixations intentionally used for control.

In this study, the classifier threshold was adjusted for achieving specificity of about 0.9, that corresponded to false alarm rate of about 0.8 per minute. This rate is far from perfect, but can be acceptable in certain situations provided that the errors can be easily corrected. For example, in a game like *EyeLines*, if it was controlled on-line with an EBCI always switched on (with no need for the switch-on button), the player could simply attempt to choose another ball if a wrong one was selected, so the wrong selection would go harmless. (To complete the move, a free cell should be selected after the selection of a ball, but spontaneous dwells on free cells are not frequent even if the player do not make any effort to refrain from them, and can be easily avoided intentionally in the case of erroneous ball selection).

The best group average value of sensitivity, among all conditions and processing schemas, was only 0.38 ± 0.14 (in one participant, it was 20, and in others, ranged 0.31 to 0.47). This value was obtained for Button fixations using Trainset 1 (only Button fixations as the target class, non-controlling fixations as the non-target class) and the committee of greedy classifiers. Such sensitivity, of course, is not sufficient if the command always should be issued using the classifier.

However, as Protzak et al. (2013) suggested, an additional gaze dwell threshold can be used to ensure issuing a command even if it was not recognized by the BCI component of the system. We observed the high similarity of the FRPs before the 500 ms from the fixation onset under the 500 ms and 1000 ms dwell threshold conditions (Figures 4, 6) and the relatively small decrease of the 500 ms trained classifier sensitivity when it was applied to the data from the 1000 ms condition (Figure 8). These results are in accordance with the recommendation by Protzak et al. (2013), demonstrating that the use of two thresholds will not likely lead to substantial drop in the classifier performance.

Classifier sensitivity followed the pattern observed for the amplitude of the negative wave that served as the marker of the controlling fixations, tending to decrease within the triplets of gaze dwells (Button–Ball–Free cell). The factors that are responsible for this decrease are yet to be determined, but it is likely that the key role was played by the order of dwells that closely followed each other. There are serious alternatives for the dwell-based EBCI in fast serial operation, such as dwell-free (Kristensson and Vertanen, 2012; Sarcar et al., 2013; Pedrosa et al., 2015) or response-based control (Publicover et al., 2016). The EBCI, thus, can be more suitable for single “clicks” or for starting a sequence, so that the rest of it will be entered using one of these alternative approaches.

Although the LDA classifier with shrinkage regularization is one of the methods of choice for ERP based BCIs (see, e.g., Blankertz et al., 2011), a simple classification approach based on the committee of greedy classifiers demonstrated at least comparable performance with our FRP data. In future studies, other classification approaches should be tested for the EBCI, especially those that were successful with similar data patterns, such as Locality Preserving Projections algorithm that provided good results for movement-related potentials (Xu et al., 2014). A relatively low intersubject variability of the negativity that marked the controlling fixations suggests that the classifier training can provide higher results when trained on the same data with priors obtained from other participants.

A thorough search for better feature sets can result in even higher improvement in performance. For example, in a preliminary study adding wavelet features extracted from the same data to the amplitude features provided significant increase of AUC up to 0.75 ± 0.04 (M ± SD for the same group) for shrinkage LDA classifier (Shishkin et al., 2016a). Note that in the current study we avoided using any data from the EEG earlier than 200 ms after fixation onset for most reliable exclusion of any contamination by artifacts, although earlier EEG samples from the fixation interval and pre-saccadic activity may include additional useful information. Moreover, our baseline choice could prevent extraction of any activity that was stable within our interval (e.g., the anterior component of SPN). Non-EEG information, especially gaze data and data about the fixated objects, their environment, possible types of current activity of the user and so on can be used as additional features, or, in some cases, for selecting a classifier (e.g., specific classifiers can be trained for different steps in sequences of actions, as in action triplets in our game). If sufficiently large amounts of gaze-synchronized EEG data will be harvested during the use of EBCIs, it will become possible to apply deep learning algorithms (LeCun et al., 2015; see also Nurse et al., 2016, on deep learning implementation at TrueNorth chip for EEG/ECoG/LFP data) that are able to find hidden patterns in the data and strongly improve classification performance.

However, it is possible that the sensitivity will not be improved to values close to 100%. What can be the added value of the BCI component if its sensitivity would be, for example, about 0.7, and the long dwells (without a BCI!) should be yet used in the rest of trials? We expect that even in this case the hybrid EBCI can provide advantage over usual gaze-based interaction when the interaction should require as little effort as possible. Indeed, the user will not need to take care about his or her attention and gaze to prevent unwanted “clicks,” to confirm the command, etc., except to be ready to fixate slightly longer in certain cases. Moreover, it is possible that many of the misses are caused by low attentional concentration. In such cases, it might be even useful to avoid too fast interface response.

It is also possible that practice will lead to more stable and higher EEG marker amplitude through the operant conditioning mechanism. Practice can improve intentional regulation of the slow cortical potentials when they are used for BCI-based communication (Neumann et al., 2004), and the same may appear to be also true for the EBCI based on SPN-like slow activity. To test this hypothesis, the FRP dynamics and EBCI performance can be studied during prolonged use of the online EBCI. The use of additional, longer dwell time threshold makes possible intensive practice even with low sensitivity of the classifier. It was thus important that the study demonstrated high similarity of the 200–500 ms interval of FRPs of controlling fixations with 500 ms and 1000 ms dwell time thresholds.

### Fast fixation-based EBCIs: an emerging class of effective hybrid BCIs

The gaze fixation and FRP data complement each other when used as an input to a hybrid interface: the fixation onset provides the temporal marker required for EEG segmentation and FRP extraction, the FRP provides features for a passive BCI classifier that automatically recognizes target finding (in the case of visual search) or issuing a command (in intentional dwell-based control), and the gaze position indicates the target (Protzak et al., 2013; Finke et al., 2016). This combination may enable fluent interaction both in visual search (Finke et al., 2016; see also Introduction) and in intentional dwell-based control (Protzak et al., 2013, and the current study).

These two types of interaction are rather different. In a visual search, the user typically makes the decision about what target he or she is looked for in advance, but the target location is not known and should be found during exploration of a scene. In intentional control, the scene and all objects in it can be well known to the user, who should be given an option not to evoke any interaction even when looking at them until the decision to interact will be made. Moreover, it is not unlikely that such an EBCI will be able to make “clicks” even at freely chosen places without any defined objects, for example, when used for drawing. While the P300 wave is important in the search tasks (Finke et al., 2016), in the intentional dwell-based interaction a negative SPN-like wave and no signs of P300 were observed (Figures 6, 7 in the current paper). Nevertheless, in both cases the BCI task is the same: to detect a target fixation among non-target fixations, in other words, just to provide “yes” or “no” answer for each fixation. Given the limitations of non-invasive brain signals and the need to use single-trial data, it is important that the BCI task is made as simple as this. Gaze single-trial data provide much more reach information, but they lack the modalities that can be extracted from brain signals. Therefore, it is natural to combine the gaze and brain state data in a multimodal (hybrid) human-machine interface (Velichkovsky and Hansen, 1996).

It seems possible that some other psychophysiological paradigms of interaction with computer GUI elements (and, possibly, with other visual media, virtual and real objects, robot parts, etc.) can be also useful for evoking brain activity that can be quickly recognized and used to act at a fixated location. Due to similarity of the input data, all such tasks can be implemented in interfaces, for the use in different situations, with the same hardware and even software. A “second-order-hybrid” BCI built in this way may provide a highly fluent and natural means of interaction. Other functions of passive BCI (Zander and Kothe, 2011) and brain state monitoring (Van Erp et al., 2012), such as based on estimation of attention level for different objects or using error potential detection, also can benefit from fixation-based EEG segmentation and can be easily incorporated into the same system to support adaptation (immediate or based on long-term data analysis) of the interface and connected machines to the user's attentive states and interests.

### Augmentation of human-computer interaction with passive dwell-based EBCIs

Although in most cases computers assist people in their mental, not physical activities, for every interaction with a computer the motor system should be used. Effects of physical workload on mental activity have not been much studied (DiDomenico and Nussbaum, 2011), and effects of light physical load on some specific kinds of mental activity, such as creative and/or highly focused, seems not been investigated at all. It is not unlikely that even a light physical load can interfere with mental work, at least in certain individuals, and that full freeing from physical activity can provide certain benefits, at least in certain cases of high focus on a mental task. In addition, certain forms of computer use, e.g., viewing artistic images, may benefit from enabling this activity in a highly relaxed state, without any use of skeletal muscles. To test the hypothesis that the non-motor interaction with computers can provide special benefits for the intellectual activities requires an interface enabling such interaction in unobtrusive way. Such an interface must have much better performance than the existing BCIs and without the burden of the Midas touch problem (or of existing solutions to it) associated with the gaze control technologies. EBCIs may become the first type of interface suitable for this.

With a tool for the separation of intentional and spontaneous fixations, potential benefits of the gaze based interaction could be high, due to the natural co-ordination between gaze and action. A user often fixates their gaze on a GUI button or a link earlier than approaches them with the mouse cursor (Huang et al., 2012; Liebling and Dumais, 2014) or before manually touching it on a touchscreen (Weill-Tessier et al., 2016). Fixating gaze at action location prior to reaching it or prior to the action is also observed when locations or objects in the physical world are reached and manipulated (Land et al., 1999; Neggers and Bekkering, 2000; Johansson et al., 2001). Gaze leadership should not be overemphasized: for known locations, the mouse typically leads the gaze, and the gaze often goes to a new location before the mouse click occurs (Liebling and Dumais, 2014), a behavior that is impossible with a dwell-based EBCI. In addition, it is not likely that it will not be possible to significantly reduce the dwell duration in dwell-based EBCIs comparing to our settings, where it was at least 500 ms plus the time of reaction to feedback. For serial operation and automatized actions, dwell-free approaches seem more relevant to optimize gaze based interaction. However, gaze dwells are a natural mean to convey intentions in social interactions, and it seems natural to use them in interaction with computers as well. What the dwell-based passive EBCI may offer is not speed but fluency of operation: it could become a tool to convert intentions into actions seamlessly and without any involvement of motor system. Ideally, such an EBCI will not require from the user anything more than just looking and willing to act.

The use of passive BCIs can be not the only way to enable such a fluent interaction. For example, for the interaction with anthropomorphic robots that could be considered as partners rather than tools, we proposed to use “joint attention” gaze patterns (gaze gesture sequences) that are developed in infancy and can be automatically used by adults. Most of our participants easily found the ways to interact with a simple model of an anthropomorphic robot which responded to such patterns, although no information was provided to them about these patterns (Fedorova et al., 2015). However, we believe that for a certain range of tasks the use of dwell-based EBCIs will be optimal.

### From the wish switch to the wish mouse

Gray Walter, who discovered the CNV, created the first BCI, the “wish switch” (Regan, 1989, p. 218) as early as in 1964. It sent a command to a projector to present the next picture when the patient was just going to press the button to advance the projector. The BCI was directly connected to the patient's motor cortex and could detect “the wish” even before the actual button press (Graimann et al., 2010). Later, Gray Walter proposed separate use of the CNV components, the Expectancy Wave (for direct control) and earlier Intention Wave (“so that the subject has the desired experience before any action has been taken”—Walter, 1966), but it seems that such experiments were never made.

Later on, slow negative potentials that could be related to expectation and intention (but possibly to other mental activities as well) were used, together with positive deflections, by Birbaumer and his colleagues in their “Thought Translation Device” (e.g., Neumann et al., 2003, 2004), and the Lateralized Readiness Potential (LRP) was used in the Berlin BCI group's early works (Blankertz et al., 2002, 2003). Computer screen color control through a BCI, that presumably responded to individual's wishes (here, unconscious ones), was demonstrated by Kaplan et al. (2005) with the use of rhythmic EEG components instead of the negative deflections. The majority of BCI studies were focused on techniques that do not respond to wishes and intents directly, such as motor imagery and responses to stimuli. Recently, slow premotor potentials were employed for developing neurorehabilitation BCIs (Niazi et al., 2011; Ibáñez et al., 2014; Lew et al., 2014; Xu et al., 2014; Jiang et al., 2015; Shakeel et al., 2015). But probably only the works by Zander's group (Ihme and Zander, 2011; Protzak et al., 2013) addressed the direct conversion of intentions into actions in line with the elegant approach by Gray Walter, now also enhanced with gaze capabilities. In the current work, we demonstrated that this combination can be efficient in realistic settings, and described an EEG marker for the controlling gaze fixations. As we discussed above, this marker is similar to SPN, the phenomenon that is again a member of the family of slow negative cortical potentials related to anticipation (Van Boxtel and Böcker, 2004).

Gaze is an ideal complement to brain activity in an interface aiming on a direct conversion of intentions into actions: it is itself driven by interest and wishes, but is able to convey the information about location, not easily available even from invasive brain signal recordings. Thus, combining eye tracking with the detection of FRP correlates of the wish to make a click at the fixated location will lead to creation of a “wish mouse,” a tool that will possibly provide unusually fluent interaction with computers both for people with motor disabilities and for healthy individuals.

## Conclusion

This study demonstrated that intentional gaze fixations for 500 ms, used to control a computer interface through eye tracking technology, can be discriminated from spontaneous gaze fixations of the same duration using only 300 ms EEG segments. This was done by recording EEG from healthy participants who played a game with real-time control by 500 ms gaze fixations and by running an offline BCI simulation. We also described an EEG marker for the controlling gaze fixations, which was prominent in all our 8 participants. The marker was a negativity with occipitoparietal focus, similar to Stimulus-Preceding Negativity (SPN) but differed from it by its lateralization. In the subsequent work, we plan to test the online version of the Eye-Brain-Computer Interface (EBCI) using this EEG marker.

## Author contributions

SS designed the study. SS, YN, AT, ES, and AF developed the methodology. AF, ES, and YN collected the data. SS, AT, ES, BK, YN, and AF analyzed the data. SS wrote the first draft. AF and SS drew the picture for Figure 1. SS and BV wrote the final draft. BV and SS supervised all aspects of the research.

## Funding

This work was partly supported by the Russian Science Foundation, grant 14-28-00234 (all parts of the work except for the literature analysis and the development of the greedy classifier committee) and by the Russian Foundation for Basic Research, grant ofi-m 15-29-01344 (the development of the greedy classifier committee).

### Conflict of interest statement

The authors declare that the research was conducted in the absence of any commercial or financial relationships that could be construed as a potential conflict of interest.
